# Correlation between ^18^F-FDG maximum standardized uptake value with CD147 expression in lung adenocarcinomas: a retrospective study

**DOI:** 10.7717/peerj.7635

**Published:** 2019-09-09

**Authors:** Fei Guo, Xueyan Li, Guodong Yao, Guangchun Zeng, Lijuan Yu

**Affiliations:** 1 Department of Radiology, Harbin Medical University Cancer Hospital, Harbin, China; 2 Department of PET/CT, Harbin Medical University Cancer Hospital, Harbin, China; 3 Department of Pathology, Harbin Medical University Cancer Hospital, Harbin, China

**Keywords:** Maximal standardized uptake values (SUVmax), ^18^F-fluorodeoxyglucose positron emission tomography (^18^F-FDG PET), Chemotherapy resistance, Cluster of differentiation 147 (CD147), Lung adenocarcinoma

## Abstract

**Background:**

The pro-tumoral action of the cluster of differentiation 147 (CD147), which is associated with the chemotherapy resistance of lung adenocarcinoma, is partly due to accelerated tumor cell glycolysis. ^18^F-fluorodeoxyglucose positron emission tomography (^18^F-FDG PET) metabolic parameters included maximal standardized uptake value (SUVmax), mean standardized uptake value (SUVmean), metabolic tumor volume (MTV), and total lesion glycolysis (TLG), which are non-invasive markers of the glucose metabolism of tumor cells in vivo. This study aimed to clarify the correlation between PET metabolic parameters and CD147 expression, and to evaluate the prognostic value of CD147 expression in resectable lung adenocarcinoma patients.

**Methods:**

A total of 89 lung adenocarcinoma chemotherapy-naive patients who underwent ^18^F-fluorodeoxyglucose positron emission tomography and computerized tomography scan before pulmonary surgery were retrospectively analyzed. The PET metabolic parameters were calculated by ^18^F-FDG PET imaging, and CD147 expression was analyzed by immunohistochemistry. SUVmax, SUVmean, MTV, and TLG compared for their performance in predicting the expression of CD147 were illustrated with statistical analysis. All patients were then followed-up for survival analysis.

**Results:**

The SUVmax was significantly correlated with the CD147 expression and was the primary predictor for the CD147 expression of lung adenocarcinoma. A cut-off value of the SUVmax, 9.77 allowed 85.1% sensitivity and 64.3% specificity for predicting the CD147 positive lung adenocarcinoma. CD147 expression was correlated with tumor differentiation and metastasis. Univariate survival analysis showed that CD147 expression was significantly associated with a shorter overall survival (OS) time. Multivariate analysis revealed that CD147 was an independent prognostic factor of lung adenocarcinoma patients.

**Conclusion:**

The SUVmax of a primary tumor measured with ^18^F-FDG PET may be a simple and non-invasive marker for predicting CD147 expression in lung adenocarcinoma. CD147 is an independent prognostic factor related to OS of postoperative lung adenocarcinoma patients.

## Introduction

Lung cancer is a leading cause of tumor-related death worldwide, and non-small cell lung cancer (NSCLC) accounts for 80–85% of lung cancer-attributed deaths (Siegel, Miller & Jemal, 2015). Lung adenocarcinoma is the most common histological subtype of NSCLC and has been afflicting an increasing proportion of patients globally (Kadara, Kabbout & Wistuba, 2012). The early diagnosis and effective therapy of lung adenocarcinoma are critical for achieving favorable long-term outcomes in patients.

Chemotherapy is the primary treatment modality for lung adenocarcinomas for both early stage and advanced-stage diseases (Zarogoulidis et al., 2013). While different chemotherapies are being used in the clinic, cisplatin-based regimens are the most frequently used in the treatment of lung adenocarcinoma as it can improve the survival in some patients (Heon & Johnson, 2012; Lwin, Riess & Gandara, 2013). However, due to the rising rates of drug resistance, the majority of patients exhibit an inadequate response to chemotherapy, which often leads to drug toxicities without any significant therapeutic benefits (D’Amato et al., 2006). Therefore, further understanding of the molecular mechanism of chemoresistance of cancer cells is imperative for designing and developing novel therapies for the treatment of lung cancer (Shanker et al., 2010).

Cluster of differentiation 147 (CD147), also known as EMMPRIN or Basigin, is a member of the immunoglobulin superfamily and a multifunctional transmembrane glycoprotein, which is highly expressed on the plasma membrane of most malignant tumors. It plays critical roles in tumor proliferation, invasion, metastasis, angiogenesis, and the development of chemoresistance (Riethdorf et al., 2006; Weidle et al., 2010; Xiong, Edwards Iii & Zhou, 2014). Recently, a growing body of evidence has suggested that the pro-tumoral action of CD147 is partly due to the interaction between two monocarboxylate transporters, MCT1 and MCT4, which accelerates tumor cell glycolysis by increased glucose uptake, lactate efflux, and the production of adenosine triphosphate (Huang et al., 2015; Li et al., 2016; Marchiq et al., 2015; Walters, Arendt & Jelinek, 2013). The elevated production of lactate within the tumor tissue leads to an acidic microenvironment, which provides the tumor cells with an invasive advantage and enables the inactivation of chemotherapeutic agents (Ganapathy-Kanniappan & Geschwind, 2013; Pelicano et al., 2006). Several studies in the past few years have suggested that CD147 is associated with the progression of tumor development and cisplatin-based chemotherapy resistance of lung adenocarcinoma and may be a potential therapeutic target (Feng et al., 2017; Yang et al., 2017; Zeng et al., 2011).

Unlike adjacent normal tissues, the majority of human tumors are characterized by a high rate of glycolysis for energy production during malignant progression (Ganapathy-Kanniappan & Geschwind, 2013; Pelicano et al., 2006). This biological characteristic of tumors has been exploited in ^18^F-fluorodeoxyglucose positron emission tomography and computerized tomography (^18^F-FDG PET/CT), which has shown important clinical value in the diagnosis, staging, planning, and therapeutic monitoring of the disease (Jadvar, Alavi & Gambhir, 2009; Kelloff et al., 2005). The ^18^F-FDG PET metabolic parameters included maximal standardized uptake value (SUVmax) was calculated using the following formula: maximum pixel value with the ROI activity/(injected dose/body weight), mean standardized uptake value (SUVmean was the averaged standardized uptake value of all pixels within ROI), metabolic tumor volume (MTV was the volume of hypermetabolic tissue with a standardized uptake value greater than the threshold value), and total lesion glycolysis (TLG was the MTV multiplied by the SUVmean of the tumor), which are non-invasive imaging markers of the glucose metabolism of tumor cells in vivo. We hypothesized that the PET metabolic parameters are correlated with CD147 expression in lung adenocarcinoma. We further hypothesized that the PET metabolic parameters might be used to determine the CD47-positive lung adenocarcinoma. Collectively, the primary purposes of this study were to examine the relationship between PET metabolic parameters and CD147 expression and to evaluate the prognostic value of CD147 expression in patients with resectable lung adenocarcinoma.

## Materials and Methods

### Study population

All patients who underwent ^18^F-FDG PET/CT scan before pulmonary surgery between January 2013 and December 2014 at Harbin Medical University Cancer Hospital were retrospectively reviewed. Patients who had all of the following were included in this study: (1) lung adenocarcinoma based on pathological diagnosis, (2) PET/CT scan no more than 2 weeks prior to surgery, (3) no biopsy, chemotherapy or radiotherapy before the PET/CT scan, (4) available tissue specimens for immunohistochemistry (IHC) staining, and (5) complete case records. After application of the inclusion criteria, 89 patients (50 male and 39 female) with a median age of 60 (range, 38–83 years) were enrolled in this study. The present study was approved by the Ethics Committee of Harbin Medical University Cancer Hospital (KY2016-14) and complied with the Declaration of Helsinki. Informed consent was obtained from all individual participants included in the study.

### ^18^F-FDG PET/CT imaging

All patients fasted for at least 4 h to ensure that the peripheral blood glucose concentration was less than 150 mg/dL prior to intravenous administration of ^18^F-FDG. Whole-body image acquisition with an integrated PET/CT scanner (Discovery ST; GE Medical systems, Milwaukee, WI, USA) was performed 60 min after injection of ^18^F-FDG (5.55–7.40 MBq/kg). After the scan range was set from the head to the mid-thigh, the unenhanced CT scan was performed with the following parameters: 140 kV, 150 mA, 0.8 s per rotation, 22.5 mm/s table speed, and 3.75 mm slice thickness. CT data were reconstructed from a 512 × 512 matrix to a 128 × 128 matrix to match the PET data and allow image fusion. The PET scan was performed with an acquisition time of 3 min per bed position in the two-dimensional mode immediately after the CT scan. PET-image datasets were reconstructed iteratively using the ordered subsets expectation maximization algorithm.

All CT, PET, and PET/CT images were loaded onto a dedicated Workstation (Advanced workstation 4.6; GE Medical Systems, Milwaukee, WI, USA). Semiquantitative analysis of PET images was performed by two independent nuclear medicine physicians in a blinded manner and any discrepancy was resolved by consensus. The metabolically active volume of the primary tumor was delineated automatically based on a threshold of 40% of the tumoral SUVmax using PET VCAR software. If the defined primary tumor margins were not appropriate, the tumoral outline was corrected manually. In our study, the automatic output results included the SUVmax, SUVmean, MTV, and TLG of the tumor.

### Immunohistochemical analysis

Immunohistochemical staining was carried out on paraffin-embedded lung adenocarcinoma sections of three μm thickness. Briefly, the sections were deparaffinized in xylene and dehydrated in graded alcohol. Antigen retrieval was carried out by microwave treatment with 0.01M citrate buffer (pH 6.0). After then, endogenous peroxidase activity was blocked with 0.5% hydrogen peroxide for 10 min. Non-specific IgG binding was blocked by incubation of sections with 1x-PBS containing 10% normal goat serum for 15 min at room temperature. The sections were subsequently incubated with the rabbit anti-CD147 monoclonal antibody (ab108317; Abcam, Cambridge, UK) at 4 °C overnight, followed by 1 h of incubation with HRP-labeled anti-rabbit IgG (SV0002; Boster Biological Technology Co., Ltd., Wuhan, China) at 37 °C. After that, the sections were exposed to the 3,3′-diaminobenzidine Kit (AR1022; Boster Biological Technology Co., Ltd, Wuhan, China) for color development and counterstained with hematoxylin.

Each immunostained section was microscopically viewed and scored by two independent observers without prior knowledge of the patient data, and any discordant cases were discussed for a consensus. CD147 was considered positive when the membranous staining was observed. The percentage of positive cells was scored as 0 (negative), 1 (1–25%), 2 (26–50%), 3 (51–75%), and 4 (76–100%). The staining intensity was scored as 0 (colorless), 1 (pale yellow), 2 (yellow), and 3 (brown). The IHC score was determined by multiplying the above two scores. For statistical analysis, negative and positive expression of CD147 were defined according to the IHC score of 0 and >0, respectively (Tian et al., 2013, 2015).

### Statistical analysis

All statistical analysis was performed using the SPSS software Version 19.0 (IBM, Chicago, IL, USA). Normally distributed continuous variables were analyzed by the Student’s *t*-test. Abnormally distributed continuous variables were analyzed by Wilcoxon rank-sum test. Dichotomous variables were analyzed by the χ^2^ test. Spearman correlation analysis was used to determine the relationship between the PET metabolic parameters and CD147 expression. Logistic stepwise regression was used to identify whether the PET metabolic parameters were the primary predictor for positive expression of CD147. Receiver operating characteristics (ROC) curve was used to determine the optimal cutoff value of the PET metabolic parameter for predicting the positive expression of CD147. Survival curves were estimated using the Kaplan–Meier method and differences in survival distributions were evaluated by the log-rank test. The Cox proportional hazards regression model of factors potentially related to survival was performed in order to identify which factors might have a significant influence on survival. *p* < 0.05 was considered statistically significant.

## Results

### Clinicopathological characteristics of patients

A total of 89 patients were registered in our study, including 39 females and 50 males, with an age range between 38 and 83 years (median age: 60 years). Among these 89 patients, 48 patients had stage I lung adenocarcinoma, 14 had stage II, and 27 had stage III disease. A total of 41 patients underwent postoperative adjuvant chemotherapy according to the NCCN criteria for NSCLC. Also, 47 patients had CD147-positive tumors and 42 patients had CD147-negative tumors, as revealed by immunohistochemical analysis. In the 89 patients, 42 tumors had a CD147 IHC score of 0, three tumors had an IHC score of 1, eight tumors had an IHC score of 2, seven tumors had an IHC score of 3, fourteen tumors had an IHC score of 4, seven tumors had an IHC score of 6, five tumors had an IHC score of 9, and three tumors had an IHC score of 12.

We analyzed CD147 expression levels in tissue samples from 89 patients with lung adenocarcinoma in relation to age, gender, tumor size, tumor differentiation, pathological N-stage, and pathological TNM-stage. As shown in Table 1, the difference in CD147 expression was not significant with respect to gender, age, tumor size, or pathological TNM-stage. Conversely, the difference in CD147 expression was significant in terms of tumor differentiation degree and pathological N-stage. Collectively, these findings indicate that CD147 expression was correlated with lung adenocarcinoma tumor differentiation and metastasis.

**Table 1:** Relationship between CD147 expression and characteristics of the patients.

Characteristics	CD147-negative (*n* = 42)	CD147-positive (*n* = 47)	χ^2^	*p*-value
Age (years)			0.330	0.566
<60	18 (42.9%)	23 (48.9%)		
≥60	24 (57.1%)	24 (51.1%)		
Gender			0.456	0.495
Male	22 (52.4%)	28 (59.6%)		
Female	20 (47.6%)	19 (40.4%)		
Tumor size (cm)			3.855	0.050
<3 cm	30 (71.4%)	24 (51.1%)		
≥3 cm	12 (28.6%)	23 (48.9%)		
Tumor differentiation			10.378	0.006
Well	16 (38.1%)	6 (12.8%)		
Moderate	15 (35.7%)	15 (31.9%)		
Poor	11 (26.2%)	26 (55.3%)		
Pathological N stage			4.484	0.034
N0	29 (69.0%)	22 (46.8%)		
N1-2	13 (31.0%)	25 (53.2%)		
Pathological TNM stage			3.767	0.152
I	27 (64.3%)	21 (44.7%)		
II	6 (14.3%)	8 (17.0%)		
III	9 (21.4%)	18 (38.3%)		

**Note:**

χ^2^ test was used to analyze the association between CD147 expression and characteristics of the patients.

### Relationship between PET metabolic parameter and CD147 expression

**Figure 1:**
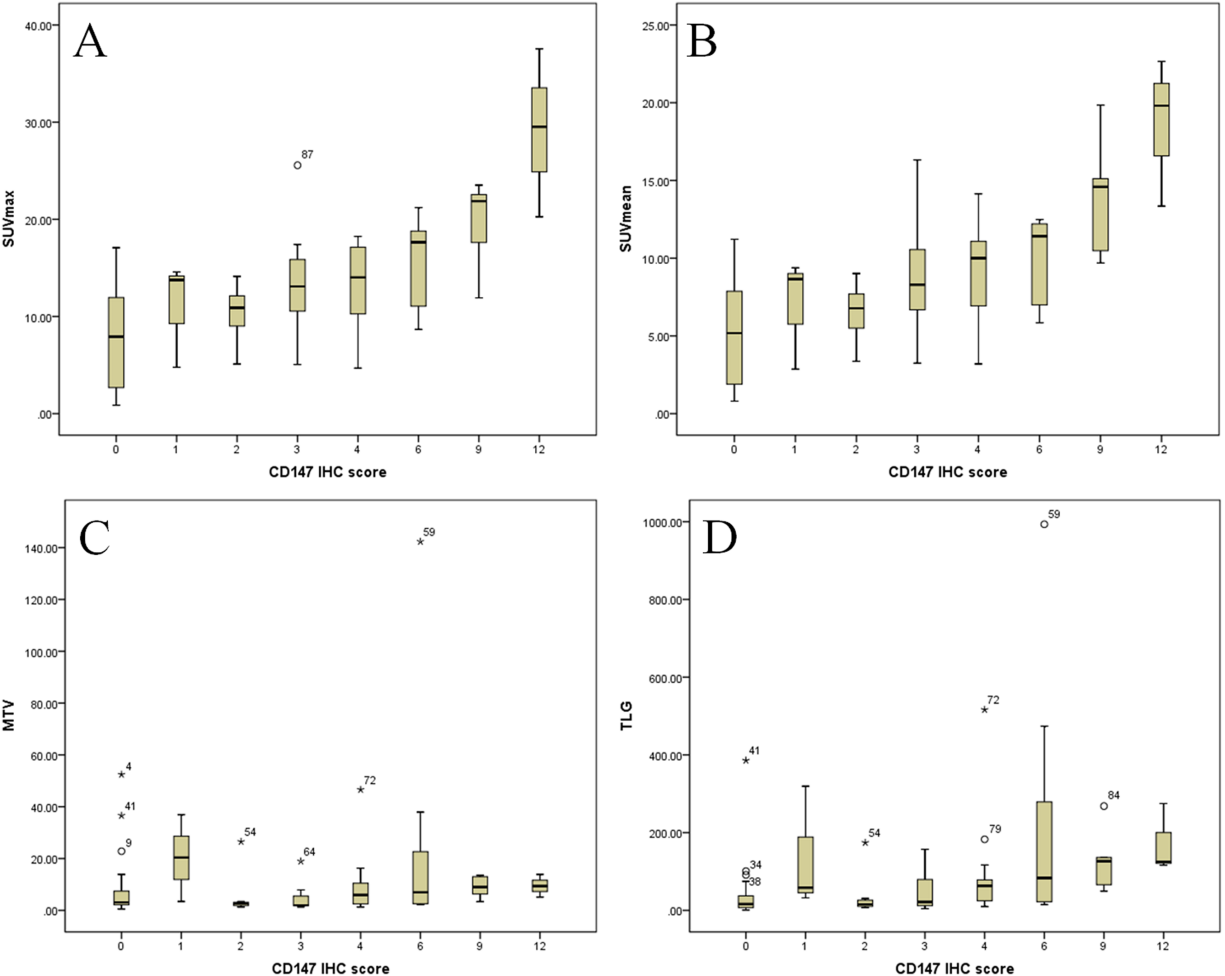
Distribution of PET metabolic parameter according to the CD147 IHC score. (A) SUVmax, (B) SUVmean, (C) MTV, (D) TLG.

According to the CD147 IHC score, the distribution of PET metabolic parameter was showed in Fig. 1. In the entire patients, the mean SUVmax was 11.37 ± 6.78, with a median value of 11.24, eight patients had a SUVmax <2.5. The mean SUVmean was 7.54 ± 4.48, with a median value of 6.98. The median MTV was 3.43, with a range of 0.49–142.35. The median TLG was 25.45, with a range of 0.89–993.34. As shown in Table 2, there was a significant difference in the SUVmax, SUVmean, and TLG between the groups with and without CD147 expression. The mean value of the SUVmax was 7.59 ± 4.91 in the CD147-negative group and 14.74 ± 6.46 in the CD147-positive group (*p* < 0.001). The mean value of the SUVmean was 5.07 ± 3.22 in the CD147-negative group and 9.74 ± 4.32 in the CD147-positive group (*p* < 0.001). The median TLG was 16.17, with a range of 0.89–385.72 in the CD147-negative group and the median TLG was 58.32, with a range of 4.23–993.34 in the CD147-positive group (*p* < 0.001). Collectively, these findings indicate that CD147 expression may be correlated with SUVmax, SUVmean, and TLG.

**Table 2:** Comparison of PET metabolic parameter according to CD147 expression.

PET metabolic parameter	CD147-negative (*n* = 42)	CD147-positive (*n* = 47)	*p*-value
SUVmax (mean ± SD)	7.59 ± 4.91	14.74 ± 6.46	<0.001
SUVmean (mean ± SD)	5.07 ± 3.22	9.74 ± 4.32	<0.001
MTV (median, range)	3.08, 0.49–52.43	5.13, 1.26–142.35	0.140
TLG (median, range)	16.17, 0.89–385.72	58.32, 4.23–993.34	<0.001

**Note:**

Abbreviations: SD, standard deviation; SUVmax, maximum standardized uptake value; SUVmean, mean standardized uptake value; MTV, metabolic tumor volume; TLG, total lesion glycolysis.

**Figure 2:**
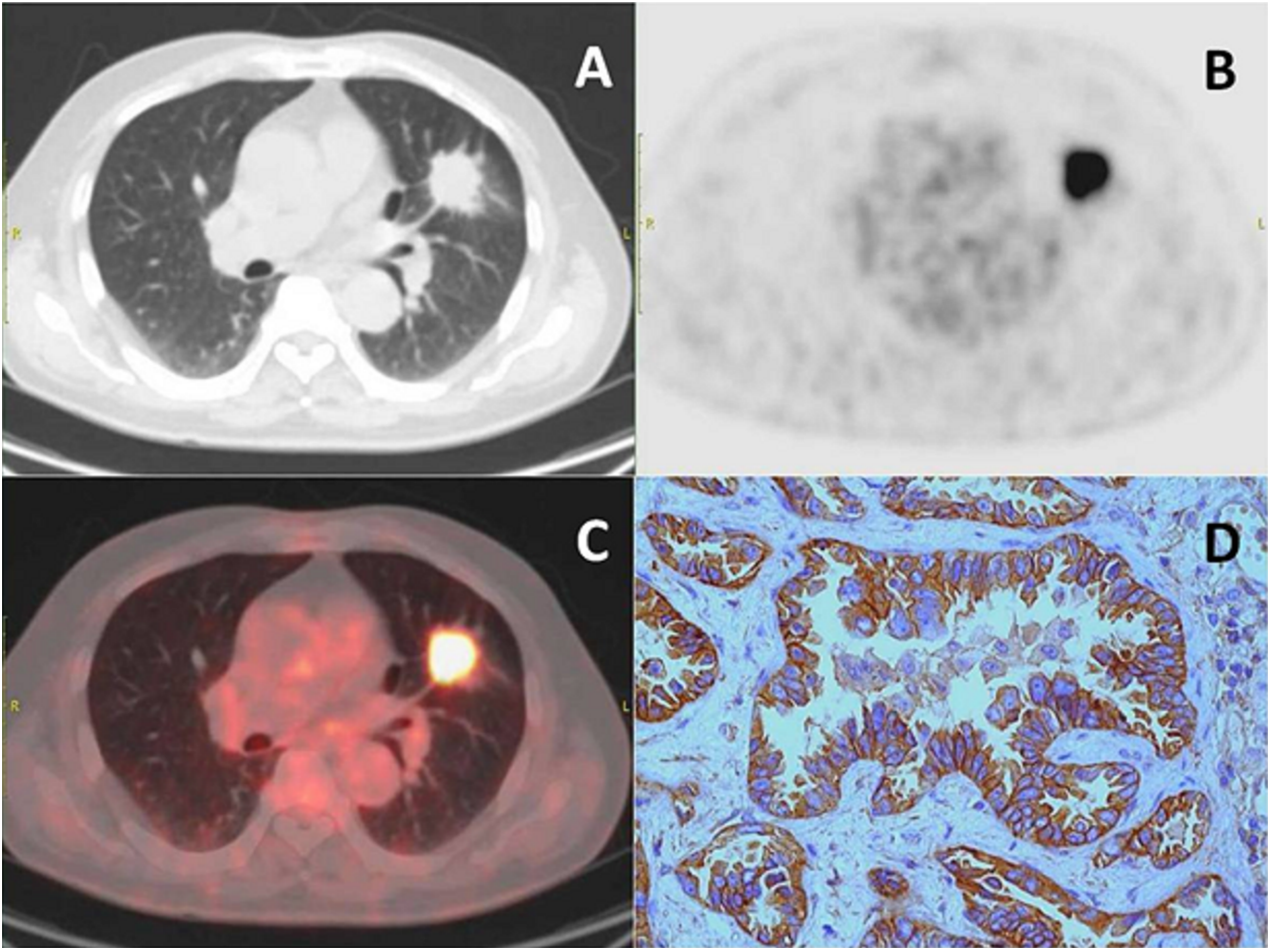
Representative images of PET-CT and immunohistochemistry. Transaxial images of (A) diagnostic CT, (B) FDG-PET, (C) merge of PET and CT images, and (D) immunohistochemical staining for CD147 (magnification, ×400). The staining intensity was scored as 3. The percentage of positive cells was scored as 4. Finally, the CD147 IHC score was 12.

**Figure 3:**
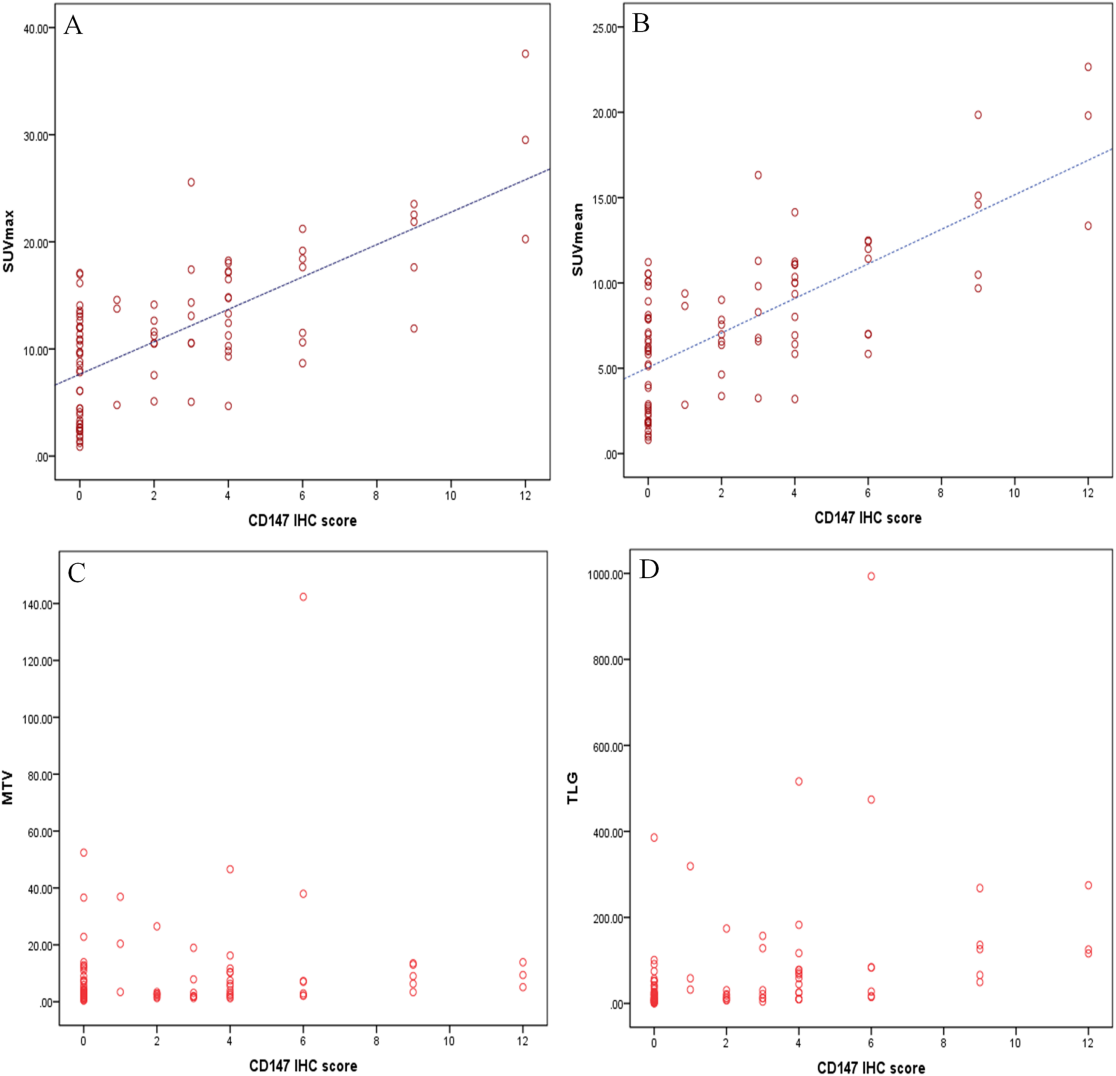
Correlation analysis between PET metabolic parameter and CD147 IHC score. (A) and (B) The SUVmax and SUVmean showed a linear correlation with the CD147 IHC score with a correlation coefficient of 0.625 and 0.630, respectively (*p* < 0.01). (C) and (D) There was no significant correlation between CD147 IHC score and MTV or TLG.

We performed the immunohistochemical analysis in order to examine the correlation between the PET metabolic parameter and the CD147 expression in lung adenocarcinoma according to the CD147 IHC score (Fig. 2). As shown in Fig. 3, CD147 IHC scores showed no significant correlation with MTV or TLG. However, the SUVmax and SUVmean showed a linear correlation with CD147 IHC scores, and revealed correlation coefficients of 0.625 and 0.630, respectively (*p* < 0.01). Also, CD147-positive tumors had significantly higher SUVmax and SUVmean than CD147-negative tumors (Table 2). Based on these findings, we hypothesized that the SUVmax and SUVmean might be valuable in predicting the CD147 expression levels in lung cancer.

### SUVmax is the primary predictor for CD147 expression

Using the logistical stepwise regression model, the CD147 IHC scores were inputted as the dependent variable, and other factors including SUVmax, SUVmean, tumor differentiation, and pathological N-stage as the independent variables. As shown in Table 3, we found that SUVmax was the primary predictor for CD147 expression in lung adenocarcinomas (*R*^2^ = 0.374, *p* = 0.023).

**Table 3:** Logistic stepwise regression analysis of primary predictor for CD147 expression.

Characteristic	β	SE	Wald χ^2^	OR (95% CI)	*p*-value
Intercept	−1.455	0.434	11.253	0.233	0.001
SUVmax	1.881	0.553	11.560	6.562 [2.218–19.410]	0.001

**Figure 4:**
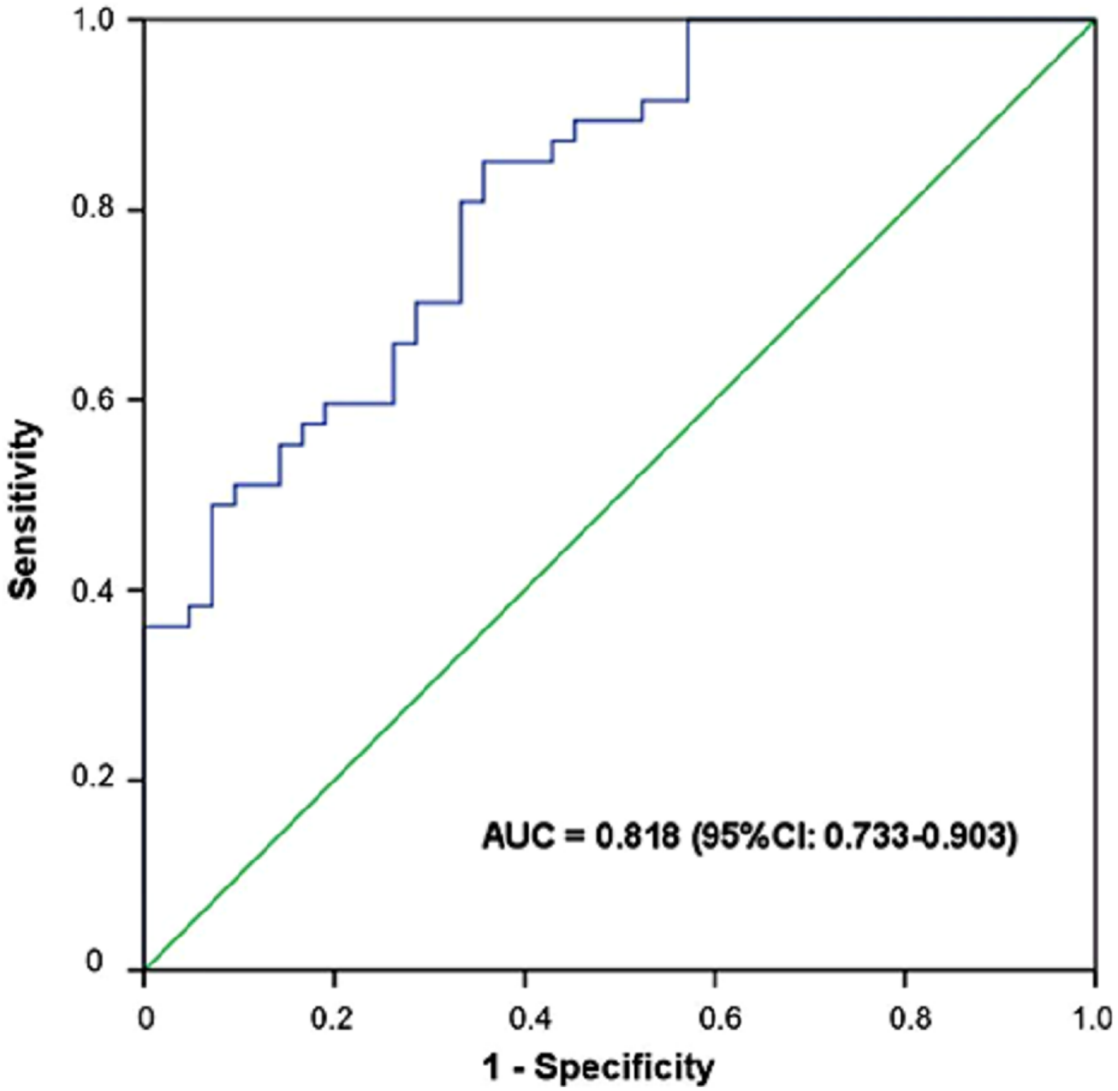
Determination of the cutoff value of SUVmax by the receiver operating characteristics (ROC) curve. The ROC curve was used to determine the optimal cutoff value of SUVmax for predicting CD147-positive lung adenocarcinoma. Area under the curve: 0.818; 95% CI: 0.733-0.903. An SUVmax of 9.77 or higher indicated that the lung adenocarcinoma was CD147-positive with a sensitivity of 85.1% and specificity of 64.3%.

The cutoff value of the SUVmax that would best predict the CD147 expression in lung adenocarcinoma was determined by ROC analysis (Fig. 4). The ROC curve analysis revealed an area under the curve of 0.818 with the 95% CI ranging from 0.733 to 0.903. The optimal cutoff value of the SUVmax was 9.77, which resulted in a sensitivity of 85.1% and a specificity of 64.3% for predicting CD147 expression (Fig. 4). Thus, the SUVmax of 9.77 was the appropriate cutoff value to predict the CD147 expression in lung adenocarcinoma with higher sensitivity and lower specificity.

### Prognostic value of CD147

**Figure 5:**
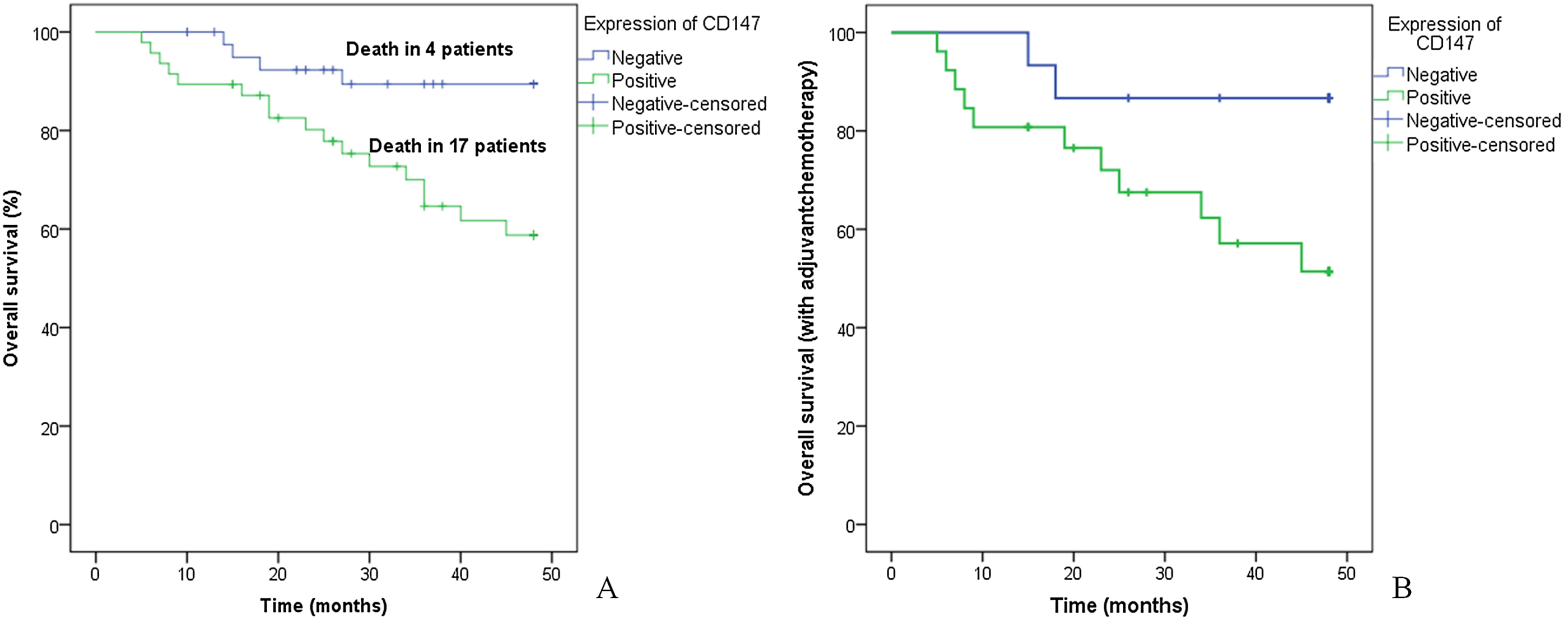
Kaplan–Meier survival curve. (A) In the entire patients, CD147-positive patients had significantly shorter overall survival than CD147-negative patients (*p* = 0.004). (B) In those who underwent postoperative chemotherapy, the CD147-positive patients had significantly shorter overall survival than CD147-negative patients (*p* = 0.045).

All patients were followed-up for survival analyses, with a median follow-up duration of 36.2 months (95% CI [33.19–39.21] months). A total of 21 patients (23.6%) died from lung adenocarcinoma during the duration of this study. Among these 89 patients, CD147-positive patients had significantly shorter overall survival (OS) than CD147-negative patients, and the median OS time was 38.12 months (95% CI [33.92–42.33]) for CD147-positive patients and 44.91 months (95% CI [42.01–47.81]) for CD147-negative patients (*p* = 0.004, Fig. 5A).

Among the 41 patients who underwent postoperative adjuvant chemotherapy, 26 patients (63.4%) were CD147 positive and 15 (36.6%) were CD147 negative. A sub-analysis of patient survival was performed for this study. CD147-positive patients had significantly shorter OS than CD147-negative patients, and the median OS time was 35.20 months (95% CI [28.77–41.63]) for CD147-positive patients and 43.80 months (95% CI [38.37–49.23]) for CD147-negative patients (*p* = 0.045, Fig. 5B). These results indicated that CD147 expression was predictive of worse clinical outcomes in postoperative lung adenocarcinoma patients.

As shown in Table 4, the univariate analysis identified five variables as having a statistically significant association with the OS of all patients, which included tumor differentiation (*p* = 0.044), pathological TNM stage (*p* = 0.024), SUVmax (*p* = 0.022), SUVmean (*p* = 0.023), and CD147 expression (*p* = 0.009). In addition, a multivariate Cox proportional hazard model showed that CD147 expression (HR = 4.26, 95% CI [1.43–12.68], *p* = 0.009) independently predicted the OS for the entire study population.

**Table 4:** Univariate and multivariate Cox regression analyses of overall survival.

Variables	Univariate analysis			Multivariate analysis		
	HR	95% CI	*p*	HR	95% CI	*p*
Age						
≥60 year vs. <60 year	1.25	[0.53–2.96]	0.616			
Gender						
Female vs. Male	1.01	[0.43–2.40]	0.977			
Tumor size						
≥3 cm vs. <3 cm	1.36	[0.58–3.21]	0.478			
Tumor differentiation						
Moderate vs. Well	2.19	[0.44–10.86]	0.336			
Poor vs. Well	4.63	[1.04–20.54]	**0.044**			
Pathological N stage						
N1-2 vs. N0	1.95	[0.82–4.63]	0.130			
Pathological TNM stage						
Stage II vs. I	0.86	[0.18–4.03]	0.842			
Stage III vs. I	2.85	[1.15–7.10]	**0.024**			
SUVmax						
High vs. Low	4.16	[1.22–14.13]	**0.022**			
SUVmean						
High vs. Low	4.12	[1.21–13.99]	**0.023**			
MTV						
High vs. Low	0.95	[0.40–2.23]	0.900			
TLG						
High vs. Low	1.83	[0.78–4.30]	0.168			
CD147 expression						
Positive vs. Negative	4.26	[1.43–12.68]	**0.009**	4.26	[1.43–12.68]	**0.009**

**Note:**

Bold *p*-values indicate statistically significant numbers.

## Discussion

In the present study, we examined the correlation between PET metabolic parameter and CD147 expression and evaluated the prognostic value of CD147 expression in postoperative lung adenocarcinoma patients. The major findings included that (1) CD147 expression was correlated with lung adenocarcinoma tumor differentiation and metastasis, (2) CD147 expression predicted a worse clinical outcome in postoperative lung adenocarcinoma patients, (3) the SUVmax was correlated with the CD147 expression of lung adenocarcinoma, (4) SUVmax was the primary predictor for the CD147-positive lung adenocarcinoma, and (5) The cutoff value of the SUVmax at 9.77 provided a reliable specificity and sensitivity for predicting the CD147-positive lung adenocarcinoma.

Most cancers rely on increased aerobic glycolysis to generate energy and metabolic intermediates, which provides tumor cells with a growth and invasion advantage. However, a high glycolytic rate increases intracellular lactic acid concentrations, which may lead to cellular acidosis and acts as a negative feedback mechanism to inhibit tumor cell glycolysis. Thus, it is necessary for cancers to export excessive lactic acid in order to maintain a high glycolytic rate. Many in vitro studies have shown that CD147 accelerates tumor cell glycolysis by facilitating lactate efflux via assisted lactic acid transporters MCT1 and MCT4 in folding, stability, cell membrane localization, and functionality (Kirk et al., 2000; Le Floch et al., 2011). In particular, a recent study by Granja et al. found that the expression of CD147, MCT1, and MCT4 was significantly upregulated in NSCLC samples compared to the adjacent non-cancerous tissues. Moreover, disruption of CD147 in three NSCLC cell lines (A549, H1975, and H292) decreased the expression and activity of MCT1 and MCT4 and diminished lactic acid export, leading to a reduction in the glycolytic rate (Granja et al., 2015). Our clinical observations were consistent with the above findings. To the best of our knowledge, the present study provided the first evidence of a correlation between CD147 expression and PET metabolic parameters evaluated by ^18^F-FDG PET/CT in patients with lung adenocarcinoma. Our data showed that the tumor SUVmax and SUVmean was positively correlated with CD147 expression, as the higher the SUVmax or SUVmean, the higher CD147 expression. Our findings further demonstrated that CD147 is involved in the enhanced glycolysis of tumor cells in vivo.

Cluster of differentiation 147 has also been identified as a mediator of chemoresistance in many tumor cell lines (Weidle et al., 2010). Correspondingly, the inhibition of CD147 expression increased the sensitivity of tumor cells to cisplatin (Wang et al., 2010; Zhu et al., 2011). In particular, Zeng et al. showed that the cisplatin-resistant lung adenocarcinoma cell line A549/DDP expressed higher levels of CD147 than cisplatin-sensitive A549 cells. Moreover, the down-regulation of CD147 by RNA interference increased the inhibitory effects of cisplatin on A549/DDP cell proliferation (Zeng et al., 2011). This study also showed an association of CD147 expression with both poor responses to cisplatin-based regimens and a poor prognosis in patients with lung adenocarcinoma (Zeng et al., 2011). Mechanistically, Toole & Slomiany (2008) proposed that CD147 caused drug resistance by stimulating the production of hyaluronan on the plasma membrane of tumor cells, which has been reported to be associated with various drug resistance in tumor cells. However, CD147 expression within tumors can be heterogeneous, and invasive procedures are needed to obtain adequate amounts of the specimen for IHC. Thus, clarifying the correlation between CD147 expression and the SUVmax determined with non-invasive metabolic imaging will be beneficial in determining the appropriate therapeutic strategy for patients with lung adenocarcinoma.

Based on our findings that the CD147 IHC score was closely related with the SUVmax, and that CD147 positive tumors had a significantly higher SUVmax, it is reasonable to hypothesize that the SUVmax may be used in predicting the levels of CD147 expression in lung adenocarcinoma. To our best knowledge, this study was the first to report SUVmax as the primary predictor for CD147 expression in lung adenocarcinoma. Furthermore, when the cutoff value of the SUVmax was set at 9.77, the sensitivity and specificity for indicating the CD147 expression were 85.1% and 64.3%, respectively. Thus, FDG-PET is a simple and non-invasive technique that can be used to determine the SUVmax, which may predict CD147-related chemotherapy resistance in lung adenocarcinomas. Only a few studies have been published on the correlation of chemotherapy-related tumor marker expression with ^18^F-FDG PET metabolic parameters in NSCLC. For example, Duan et al. (2013) found that the SUVmax of primary tumor could be used for predicting p53-related chemotherapy resistance in NSCLC. Actually, it is worth noting that there are differences in glucose metabolism between lung adenocarcinoma and squamous cell carcinoma. Thus, ^18^FDG-PET should be interpreted in relationship to the histological subtypes of NSCLC (Schuurbiers et al., 2014). However, the mechanisms leading to the chemotherapy resistance of lung cancer are complicated and believed to be multifactorial, which includes increased drug efflux, drug inactivation, and sequestration by enzymes, DNA repair, target modifications, and apoptotic defects (Shanker et al., 2010). Also, using CD147 as a sole therapeutic target and the biomarker for chemotherapy resistance in lung adenocarcinoma warrants to be further corroborated. Thus, caution should be taken when using the SUVmax as an alternative marker for CD147-related chemotherapy resistance, and more bench studies and clinical trials are needed before the SUVmax can be used as a reliable marker to determine the chemotherapy resistance of lung adenocarcinomas in the clinic.

## Limitations

The present study had several limitations. First, the data were collected from a single center. Secondly, this was a retrospective study with relatively small sample size. Thirdly, multiple molecular markers of chemotherapy resistance should be included because of the complexity of lung adenocarcinoma chemotherapy resistance. Fourth, although we asked patients to slow their breathing to minimize the effect of irregular breathing on the image quality prior to the initiation of examination, we could not completely rule out the effect that breathing may have on SUV measurements.

## Conclusions

Maximal standardized uptake value is associated with and the primary predictor for CD147 expression in lung adenocarcinomas. CD147 was an independent prognostic factor related to the OS of postoperative lung adenocarcinoma patients.

## Supplemental Information

10.7717/peerj.7635/supp-1 Supplemental Information 1 Raw data of PET metabolic parameters, CD147 IHC scores of tumors, clinicopathological characteristics and survival states of patients.

10.7717/peerj.7635/supp-2 Supplemental Information 2 Codebook for the raw data.

## Additional Information and Declarations

### Data Availability

The following information was supplied regarding data availability: The raw measurements are available in the Supplemental File.
