# Supplementary material for: Correlation between 18F-FDG maximum standardized uptake value with CD147 expression in lung adenocarcinomas: a retrospective study
Source: PeerJ. 2019 Sep 9;7:e7635. doi: 10.7717/peerj.7635 (PMC6741284; doi:10.7717/peerj.7635)
Supplement: Supplemental Information 2 [file peerj-07-7635-s002.docx]

|  | 0 | 1 | 2 |
| --- | --- | --- | --- |
| Age | < 60 | ≥ 60 |  |
| Gender | Male | Female |  |
| Tumor size | < 3 | ≥ 3 |  |
| Tumor differentiation | Well | Moderate | Poor |
| Pathological N-stage | N0 | N1-2 |  |
| Pathological TNM-stage | Stage I | Stage II | Stage III |
| Expression of CD147 | Negative | Positive |  |
| SUVmax | <9.77 low | ≥9.77 high |  |
| SUVmean | <6.31 low | ≥6.31 high |  |
| MTV | <4.02 low | ≥4.02 high |  |
| TLG | <54.73 low | ≥54.73 high |  |
| Survival /Death | Survival | Death |  |
